# Enhanced eryptosis contributes to anemia in lung cancer patients

**DOI:** 10.18632/oncotarget.7286

**Published:** 2016-02-09

**Authors:** Rosi Bissinger, Carla Schumacher, Syed M. Qadri, Sabina Honisch, Abaid Malik, Friedrich Götz, Hans-Georg Kopp, Florian Lang

**Affiliations:** ^1^ Department of Physiology, University of Tübingen, Tübingen, Germany; ^2^ Department of Internal Medicine, University of Tübingen, Tübingen, Germany; ^3^ Department of Pathology and Molecular Medicine, McMaster University, Hamilton, Canada; ^4^ Department of Microbial Genetics, University of Tübingen, Tübingen, Germany

**Keywords:** anemia, eryptosis, ceramide, cell shrinkage, carcinoma

## Abstract

**Objectives:**

Anemia is a common complication of malignancy, which could result from either compromised erythropoiesis or decreased lifespan of circulating erythrocytes. Premature suicidal erythrocyte death, characterized by cell shrinkage and phosphatidylserine (PS) externalization, decreases erythrocyte lifespan and could thus cause anemia. Here, we explored whether accelerated eryptosis participates in the pathophysiology of anemia associated with lung cancer (LC) and its treatment.

**Methods:**

Erythrocytes were drawn from healthy volunteers and LC patients with and without cytostatic treatment. PS exposure (annexin V-binding), cell volume (forward scatter), cytosolic Ca^2+^ (Fluo3 fluorescence), reactive oxygen species (ROS) production (DCFDA fluorescence) and ceramide formation (anti-ceramide antibody) were determined by flow cytometry.

**Results:**

Hemoglobin concentration and hematocrit were significantly lower in LC patients as compared to healthy controls, even though reticulocyte number was higher in LC (3.0±0.6%) than in controls (1.4±0.2%). The percentage of PS-exposing erythrocytes was significantly higher in LC patients with (1.4±0.1%) and without (1.2±0.3%) cytostatic treatment as compared to healthy controls (0.6±0.1%). Erythrocyte ROS production and ceramide abundance, but not Fluo3 fluorescence, were significantly higher in freshly drawn erythrocytes from LC patients than in freshly drawn erythrocytes from healthy controls. PS exposure of erythrocytes drawn from healthy volunteers was significantly more pronounced following incubation in plasma from LC patients than following incubation in plasma from healthy controls.

**Conclusion:**

Anemia in LC patients with and without cytostatic treatment is paralleled by increased eryptosis, which is triggered, at least in part, by increased oxidative stress and ceramide formation.

## INTRODUCTION

Anemia is a common clinical condition in malignancy [1], which may result from blood loss [1], impaired erythropoiesis due to inadequate production or efficacy of erythropoietin [2, 3], decreased availability of iron [1, 4, 5] or from decreased life span of circulating erythrocytes [6]. The anemia is further confounded by the effects of cytostatic treatment [7, 8].

In theory, anemia of malignancy could result from accelerated suicidal cell death or eryptosis [6], which is characterized by cell shrinkage [9] and cell membrane scrambling with exposure of phosphatidylserine at the cell surface [10]. Phosphatidylserine exposing erythrocytes are recognized by phagocytosing cells, engulfed and thus cleared from circulating blood [9, 10].

Signaling eventually leading to eryptosis includes oxidative stress [9, 11–13], increase of cytosolic Ca^2+^ activity ([Ca^2+^]_i_) [14], ceramide formation [9], energy depletion [15] and caspase activation [9]. Eryptosis may be triggered by activation of casein kinase 1α, CDK4, or p38 kinase and by pharmacological or genetic knockout of AMP-activated kinase AMPK, cGMP-dependent protein kinase as well as sorafenib- and sunitinib-sensitive kinases [9].

Eryptosis is enhanced in several clinical conditions, such as sepsis, malaria, sickle cell disease, Wilson's disease, iron deficiency, metabolic syndrome, diabetes, hepatic, cardiac and renal failure, Hemolytic Uremic Syndrome, as well as dehydration [9, 10, 16–18]. According to animal experiments, eryptosis is similarly upregulated in malignancy [6].

The present study explored, whether and how eryptosis is enhanced in lung cancer (LC). To this end, blood was drawn from patients with LC with and without cytostatic treatment and from healthy volunteers and phosphatidylserine surface abundance, cell volume, reactive oxygen species (ROS), [Ca^2+^]_i_, and ceramide abundance determined.

## RESULTS

The present study explored whether patients with lung cancer suffer from anemia and, if so, whether the anemia could be partially due to enhanced eryptosis. Table 1 lists the clinical characteristics including the age, sex and diagnosis for each patient. Table 2 provides various blood chemistry parameters from patients without (Group I) and with (Group II) cytostatic treatment as well as healthy volunteers.

**Table 1:** Clinical diagnosis of patients studied, with and without cytostatic treatment

Number	Age	Sex	Clinical diagnosis	Treatment
1	63	m	Small cell lung cancer, Neuroendocrine carcinoma (T4 N3 M1b, stage IV, UICC/AJCC)	Carboplatin/Etoposide
2	66	m	Non small cell lung cancer, poorly differentiated adeno-carcinoma (T4 N3 M1b, stage IV, UICC/AJCC)	Cisplatin/Gemcitabine Carboplatin
3	52	m	Non small cell lung cancer, Squamous cell carcinoma (cT2a cN2 cM0, stage IIIA, UICC/AJCC)	Cisplatin/Vinorelbine Docetaxel
4	52	f	Non small cell lung cancer, poorly differentiated adenocarcinoma (cT2b cN2-3 M1b, stage IV, UICC/AJCC)	Cisplatin/Gemcitabine
5	62	m	Non small cell lung cancer, poorly differentiated squamous cell carcinoma (pT3 pN0 L0 V0 Pn0 R0, stage IIB, AJCC/UICC)	Cisplatin/Vinorelbine
6	56	m	Non small cell lung cancer, Adenocarcinoma (cT4 cN3 M1a, stage IV, AJCC/UICC)	Docetaxel
7	64	m	Small cell lung cancer (cT4 N3 M1b, stage IV, UICC/AJCC)	Carboplatin/Etoposide Topotecan
8	66	f	Non small cell lung cancer (stage IV, UICC/AJCC)	Carboplatin/Gemcitabine
9	53	f	Non small cell lung cancer, Squamous cell carcinoma (cT3 N3 M1, stage IV, UICC/AJCC)	Carboplatin/Vinorelbine Pembrolizumab
10	77	m	Non small cell lung cancer, poorly differentiated adenocarcinoma T4 N3 M1b, stage IV (UICC/AJCC)	Carboplatin/Gemcitabine
11	81	f	Small cell lung cancer, (cT2b cN2 M0, stage IIIA)	Carboplatin/Etoposide
12	60	m	Small cell lung cancer, (T4 N3 M1, stage IV)	No cytostatic treatment
13	71	f	Non small cell lung cancer, adenocarcinoma G3	No cytostatic treatment
14	74	m	Non small cell lung cancer, poorly differentiated squamous cell carcinoma (T3 cN0-1 M0)	No cytostatic treatment
15	59	m	Non small cell lung cancer, adenocarcinoma, (cT2a pN 1-2 M0)	No cytostatic treatment
16	63	f	Non small cell lung cancer, squamous cell carcinoma (cT4 cN3 cM0)	No cytostatic treatment
17	85	m	Non small cell lung cancer, Adenocarcinoma (cT3-4 cN0-1 M0)	No cytostatic treatment

**Table 2:** Characteristics of patients and healthy volunteers

	Healthy volunteers (A)	Patients without cytostatic treatment (Pat. Group I) (B)	Patients with cytostatic treatment (Pat. Group II) (C)	*P*-value
**Mean age (years)**	57.1 ± 1.8	68.7 ± 4.1	63.0 ± 2.9	0.0073** (A vs. B) 0.0839 (A vs. C) 0.2722 (B vs. C)
**Sex**	11 males 4 females	4 males 2 females	7 males 4 females	
**Plasma creatinine concentration** (mg/100 ml)	0.5-0.8 (female) 0.6-1.1 (male)	0.6 ± 0.04	0.8 ± 0.07	0.1159 (B vs. C)
**Plasma uric acid** (mg/100 ml)	2.4-5.7 (female) 3.4-7.0 (male)	5.4 ± 0.7	5.7 ± 0.5	0.7789 (B vs. C)
**Plasma ferritin concentration** (μg/100 ml)	1.0-20 (female) 3.0-30 (male)	n.a.	44 ± 26.4	n.a.
**Red blood cell distribution width** (%)	< 14	14.0 ± 1.2	16.8 ± 0.7	0.059 (B vs. C)
**Erythrocytes** **(x10^6^/μl)**	5.2 ± 0.1	4.2 ± 0.2	3.1 ± 0.1	<0.001*** (A vs. B) <0.001*** (A vs. C) 0.001## (B vs. C)
**Hematocrit (%)**	43.6 ± 0.9	37.0 ± 2.0	28.7 ± 1.4	0.0018** (A vs. B) <0.001*** (A vs. C) 0.0027## (B vs. C)
**Hemoglobin (g/dl)**	14.5 ± 0.4	12.4 ± 0.7	9.6 ± 0.4	0.0103* (A vs. B) <0.001*** (A vs. C) 0.0029## (B vs. C)
**Reticulocytes (%)**	1.4 ± 0.1	1.8 ± 0.3	3.0 ± 0.6	0.381 (A vs.B) 0.0123* (A vs. C) 0.1625 (B vs. C)
**Mean Corpuscular Volume (fl)**	84.7 ± 1.4	88.8 ± 1.2	91.5 ± 1.0	0.0929 (A vs. B) 0.0011**(A vs. C) 0.1184 (B vs. C)
**MCH** (pg/erythrocyte)	27.0-34.0	29.7 ± 0.6	30.6 ± 0.3	0.175 (B vs. C)
**MCHC** (g/100 ml)	32.0-36.0	33.4 ± 0.4	33.4 ± 0.3	0.9094 (B vs. C)
**Plasma total protein** (g/100 ml)	6.5-8.5	7.4 ± 0.2	7.0 ± 0.1	0.2007 (B vs. C)
**C-reactive protein** (mg/100 ml)	< 0.5	4.4 ± 2.4	3.0 ± 1.1	0.5253 (B vs. C)
**Leucocytes** (/μl)	3800-10300	7607 ± 776.8	8839 ± 3285	0.7901 (B vs. C)
**Lymphocytes** **(%)**	20-45	19.2 ± 2.1	23.9 ± 5.4	0.5825 (B vs. C)
**Monocytes** **(%)**	2-8	8.2 ± 0.8	7.0 ± 1.1	0.4664 (B vs. C)
**Thrombocytes** (x 10^3^/μl)	150-450	317 ± 52.9	277.9 ± 43.2	0.5878 (B vs. C)

Values of healthy individuals and patients are given as arithmetic mean ± SEM. Values without SEM in Table 2 reflect standard clinical reference values and do not represent data from healthy controls. * (*P*<0.05), ** (*P*<0.01), *** (*P*<0.001) indicates significant difference from the healthy control group; ## (*P*<0.01) indicates significant difference from Pat. Group I.

**Figure 1:**
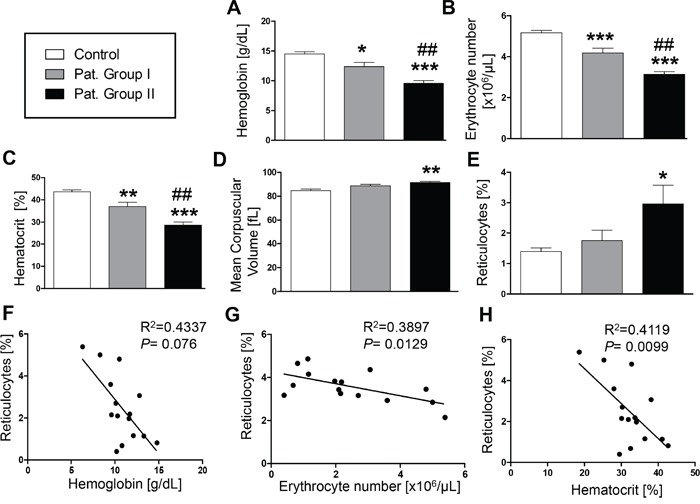
Effect of lung cancer on erythrocyte blood parameters **A–E.** Arithmetic means ± SEM of (A) hemoglobin concentration, (B) erythrocyte count, (C) hematocrit, (D) mean corpuscular volume and (E) percentage of reticulocytes in blood drawn from healthy volunteers (n=15, white bars) and patients without (n=6, gray bars) and with (n=11, black bars) cytostatic treatment. * (*P*<0.05), ** (*P*<0.01) and *** (*P*<0.001) significant difference from healthy volunteers (unpaired *t* test), ## (*P*<0.01) significant difference from patients without cytostatic treatment (unpaired *t* test). **F-H.** Reticulocyte number as a function of (F) hemoglobin concentration (g/dL) (*P*=0.076, R^2^=0.4337), (G) erythrocyte number (*P*=0.0129, R^2^=0.3897), and (H) hematocrit (*P*= 0.0099, R^2^= 0.4119) in lung cancer patients.

As illustrated in Figure 1, all LC patients in Group II had a hemoglobin level lower than 11 g/dl, an erythrocyte count of less than 3.5×10^3^/μl, and/or a packed cell volume lower than 34%, and were, thus, anemic. Group I had a significantly higher hemoglobin level (Figure 1A, 29.2%), a higher erythrocyte count (Figure 1B, 35.5%) and a higher packed cell volume (Figure 1C, 28.9%) as compared to Group II. However, all three parameters were still significantly lower in Group I as compared to the healthy volunteers. None of the healthy individuals was anemic. Mean corpuscular volume was slightly but significantly enhanced in LC patients receiving cytostatic treatment (Figure 1D, 8.0% versus controls). As shown in Figure 1E, the anemia of the patients was paralleled by significantly enhanced reticulocytosis in Group II (114.3% versus controls), an observation pointing to enhanced erythrocyte turnover. Furthermore, a negative correlation was observed between the percentage of reticulocytes in LC patients and hemoglobin level, erythrocyte count, as well as packed cell volume (Figure 1F, 1G and 1H).

**Figure 2:**
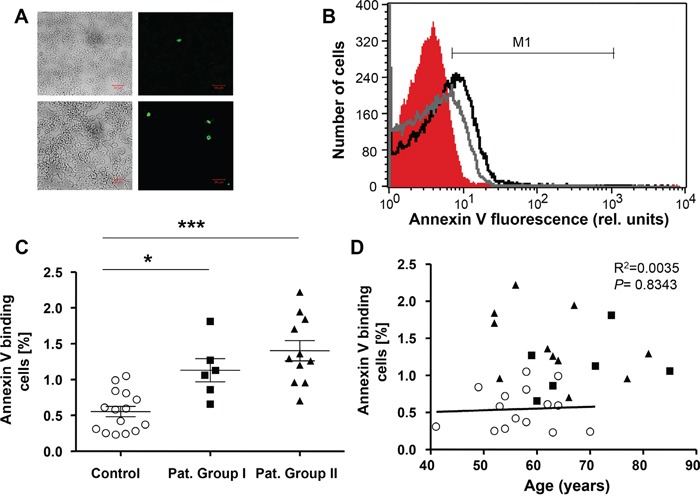
Phosphatidylserine exposure of erythrocytes drawn from patients without and with cytostatic treatment and healthy volunteers **A.** Confocal images of FLUOS-dependent fluorescence and light microscopy of human erythrocytes stained with FLUOS-conjugated annexin V. The specimens were obtained from healthy volunteers (Control; upper panels) and from lung cancer patients without cytostatic treatment (group I; lower panels). **B.** Original representative histogram of annexin V binding of erythrocytes in freshly drawn blood from healthy volunteers (red shadow) and from patients without (gray line) and with (black line) cytostatic treatment. M1 indicates the annexin V fluorescence defining the percentage of annexin V binding erythrocytes. **C.** Arithmetic means ± SEM of the percentage of annexin V-binding erythrocytes in freshly drawn blood from healthy volunteers (n=15, Control, open circles) and patients without (n=6, closed squares) and with (n=11, closed triangles) cytostatic treatment. * (*P*<0.05) and *** (*P*<0.001) significant difference from healthy volunteers (Mann Whitney test). **D.** Percentage of Annexin V binding erythrocytes in healthy individuals (open circles) and lung cancer patients of group I (n=6, closed squares) and group II (n=11, closed triangles) as a function of age. The regression line shown is calculated for healthy individuals (*P*=0.8343, R^2^=0.0035).

Further analysis addressed the possibility that the anemia was paralleled by and possibly due to accelerated eryptosis. In order to identify PS exposing erythrocytes, confocal microscopy was employed to detect annexin V positive erythrocytes. As depicted in Figure 2A, the number of annexin V positive erythrocytes was higher in freshly drawn blood from LC patients of Group I as compared to blood drawn from healthy volunteers. PS exposure was subsequently quantified in FACS analysis utilizing binding of FITC-labeled Annexin V. As shown in Figure 2B and 2C, the percentage of erythrocytes binding annexin V was significantly higher in freshly drawn blood from Group I (by 95.9%) and Group II (by 135.7%) as compared to blood drawn from healthy volunteers. As illustrated in Figure 2D, the percentage of annexin V binding cells was higher in lung cancer patients than in healthy volunteers irrespective of age.

**Figure 3:**
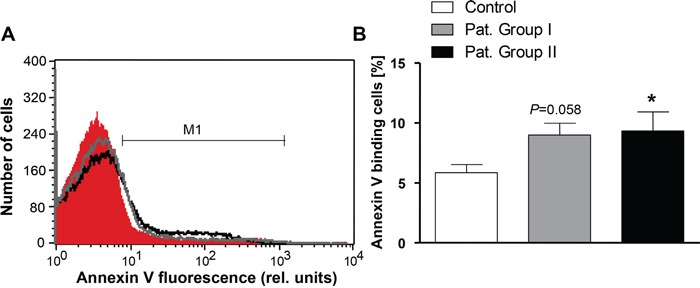
Effect of patient plasma on phosphatidylserine exposure in erythrocytes from healthy volunteers **A**. Original representative histogram of annexin V binding of erythrocytes from healthy volunteers following a 24 h exposure to plasma from healthy volunteers (red shadow) and to plasma from patients without (gray line) and with (black line) cytostatic treatment. M1 indicates the annexin V fluorescence defining the percentage of annexin V binding erythrocytes. **B.** Arithmetic means ± SEM of the percentage of annexin V-binding erythrocytes from healthy volunteers following a 24 h exposure to plasma from healthy volunteers (n=15, white bar) and to plasma from patients without (n=6, gray bar) and with (n=11, black bar) cytostatic treatment. * (*P*<0.05) significant difference from healthy volunteers (unpaired *t* test).

In order to test whether eryptosis was stimulated by a blood-borne component, erythrocytes from healthy volunteers were incubated in plasma drawn from either patients or healthy volunteers. As a result, the percentage of PS exposing erythrocytes drawn from healthy individuals tended to be higher following incubation in plasma from Group I and was significantly increased (by 59.6%) following incubation in plasma from Group II (Figure 3A and 3B).

**Figure 4:**
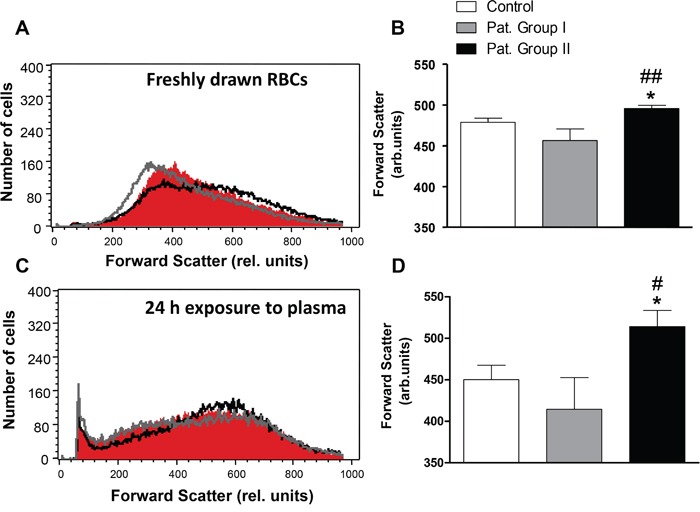
Effect of lung cancer on erythrocyte forward scatter **A.** Original representative histogram of forward scatter of erythrocytes in freshly drawn blood from healthy volunteers (red shadow) and from patients without (gray line) and with (black line) cytostatic treatment. **B.** Arithmetic means ± SEM of forward scatter geometric mean of erythrocytes in freshly drawn blood from healthy volunteers (n=15, white bar) and from patients without (n=6, gray bar) and with (n=11, black bar) cytostatic treatment. * (*P*<0.05) significant difference from healthy volunteers. ## (*P*<0.01) significant difference from patients without cytostatic treatment. **C.** Original representative histogram of forward scatter of erythrocytes from healthy volunteers following a 24 h exposure to plasma from healthy volunteers (n=15, red shadow) and to plasma from patients without (gray line) and with (black line) cytostatic treatment. **D.** Arithmetic means ± SEM of forward scatter geometric mean of erythrocytes from healthy volunteers following a 24 h exposure to plasma from healthy volunteers (n=15, white bar) and to plasma from patients without (n=6, gray bar) and with (n=11, black bar) cytostatic treatment. * (*P*<0.05) significant difference from healthy volunteers. # (*P*<0.05) significant difference from patients without cytostatic treatment (unpaired *t* test).

In order to estimate cell volume, forward scatter was determined by flow cytometry. As illustrated in Figure 4, the average forward scatter of freshly drawn erythrocytes from Group I as well as the forward scatter of healthy erythrocytes exposed for 24 h to plasma from Group I tended to be lower as compared to the average forward scatter of erythrocytes freshly drawn from healthy volunteers or erythrocytes exposed for 24 h to plasma from healthy volunteers. The average forward scatter of freshly drawn erythrocytes from Group II or exposed for 24 h to plasma from Group II, however, was slightly but significantly larger (by 3.5% in freshly drawn erythrocytes and by 14.2% in plasma) as compared to erythrocytes from healthy volunteers and those which were exposed for 24 h to plasma from healthy volunteers (Figure 4).

**Figure 5:**
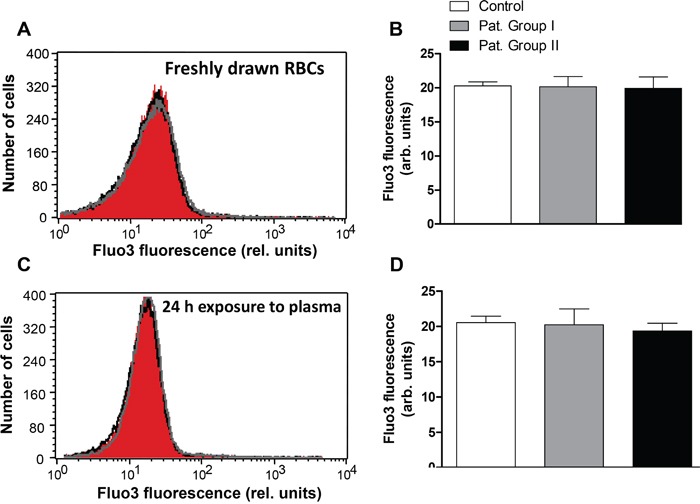
Effect of lung cancer on intracellular Ca^2+^ activity of erythrocytes **A.** Original representative histogram of Fluo3 fluorescence of erythrocytes in freshly drawn blood from healthy volunteers (red shadow) and from patients without (gray line) and with (black line) cytostatic treatment. **B.** Arithmetic means ± SEM of Fluo3 fluorescence of erythrocytes in freshly drawn blood from healthy volunteers (n=15, white bar) and from patients without (n=6, gray bar) and with (n=11, black bar) cytostatic treatment. **C.** Original representative histogram of Fluo3 fluorescence of erythrocytes from healthy volunteers following a 24 h exposure to plasma from healthy volunteers (red shadow) and to plasma from patients without (gray line) and with (black line) cytostatic treatment. **D.** Arithmetic means ± SEM of Fluo3 fluorescence of erythrocytes from healthy volunteers following a 24 h exposure to plasma from healthy volunteers (n=15, white bar) and to plasma from patients without (n=6, gray bar) and with (n=11, black bar) cytostatic treatment (unpaired *t* test).

Triggers of eryptosis include increase in erythrocyte Ca^2+^ concentration ([Ca^2+^]_i_). Accordingly, Fluo3 fluorescence was employed to quantify [Ca^2+^]_i_ in freshly drawn blood from patients and healthy volunteers. As illustrated in Figure 5A and 5B, the Fluo3 fluorescence in erythrocytes freshly drawn was not significantly different between healthy volunteers and patients from Group I and Group II. Moreover, Fluo3 fluorescence in erythrocytes exposed for 24 h to plasma from Group I or Group II was not significantly different to Fluo3 fluorescence in erythrocytes exposed for 24 h to plasma from healthy volunteers (Figure 5C and 5D).

**Figure 6:**
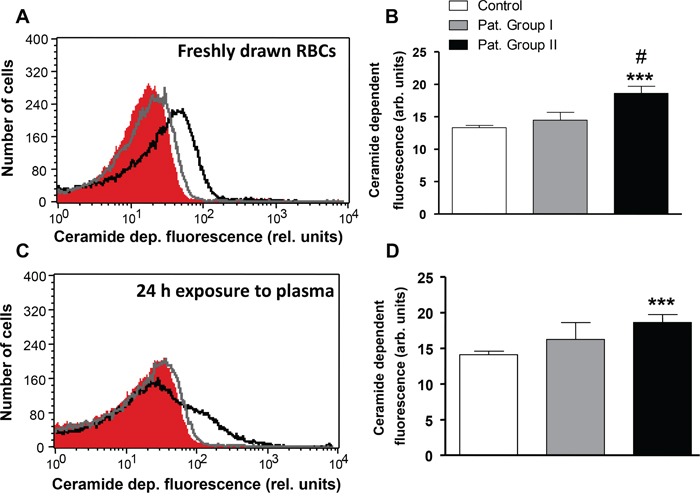
Effect of lung cancer on erythrocyte ceramide abundance **A.** Original representative histogram of ceramide-dependent FITC fluorescence of erythrocytes in freshly drawn blood from healthy volunteers (red shadow) and from patients without (gray line) and with (black line) cytostatic treatment. **B.** Arithmetic means ± SEM of ceramide-dependent FITC fluorescence of erythrocytes in freshly drawn blood from healthy volunteers (n=15, white bar) and from patients without (n=6, gray bar) and with (n=11, black bar) cytostatic treatment. *** (*P*<0.001) significant difference from healthy volunteers. # (*P*<0.05) significant difference from patients without cytostatic treatment. **C.** Original representative histogram of ceramide-dependent FITC fluorescence of erythrocytes from healthy volunteers following a 24 h exposure to plasma from healthy volunteers (red shadow) and to plasma from patients without (gray line) and with (black line) cytostatic treatment. **D.** Arithmetic means ± SEM of ceramide-dependent FITC of erythrocytes from healthy volunteers following a 24 h exposure to plasma from healthy volunteers (n=15, white bar) and to plasma from patients without (n=6, gray bar) and with (n=11, black bar) cytostatic treatment. *** (*P*<0.001) significant difference from healthy volunteers (unpaired *t* test).

Stimulators of cell membrane scrambling without increase of [Ca^2+^]_i_ include ceramide. Thus, an additional series of experiments explored, whether ceramide abundance at the erythrocyte surface is different in patients and in healthy volunteers. Ceramide abundance was visualized utilizing FITC-labeled antibodies. As illustrated in Figure 6A and 6B, the ceramide abundance tended to be higher in erythrocytes drawn from Group I and was significantly higher (by 39.9 %) in erythrocytes drawn from Group II in comparison to erythrocytes drawn from healthy volunteers. In order to test whether ceramide abundance is induced by a blood-borne component, erythrocytes from healthy volunteers were incubated in plasma drawn from either, patients or healthy volunteers. As a result, the ceramide abundance in erythrocytes following 24 h exposure to plasma from Group I tended to be higher and was significantly increased (by 31.7%) following exposure to plasma from Group II in comparison to erythrocytes exposed to plasma from healthy volunteers (Figure 6C and 6D).

**Figure 7:**
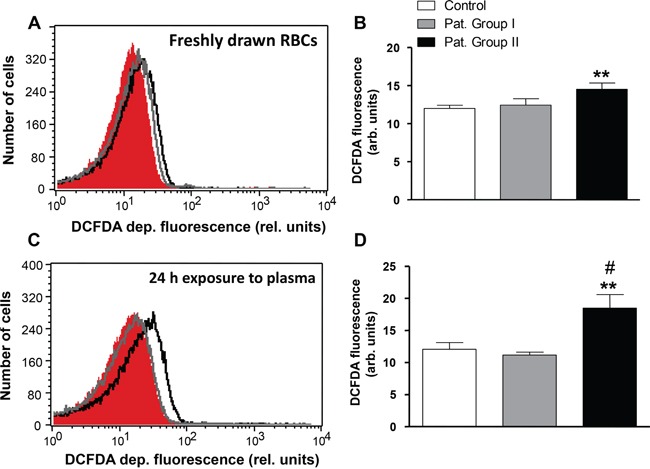
Effect of lung cancer on generation of reactive oxygen species in erythrocytes **A.** Original representative histogram of DCFDA fluorescence of erythrocytes in freshly drawn blood from healthy volunteers (red shadow) and from patients without (gray line) and with (black line) cytostatic treatment. **B.** Arithmetic means ± SEM of DCFDA fluorescence of erythrocytes in freshly drawn blood from healthy volunteers (n=15, white bar) and from patients without (n=6, gray bar) and with (n=11, black bar) cytostatic treatment. ** (*P*<0.01) significant difference from healthy volunteers. **C.** Original representative histogram of DCFDA fluorescence of erythrocytes from healthy volunteers following a 24 h exposure to plasma from healthy volunteers (red shadow) and to plasma from patients without (gray line) and with (black line) cytostatic treatment. **D.** Arithmetic means ± SEM of DCFDA fluorescence of erythrocytes from healthy volunteers following a 24 h exposure to plasma from healthy volunteers (n=15, white bar) and to plasma from patients without (n=6, gray bar) and with (n=11, black bar) cytostatic treatment. ** (*P*<0.01) significant difference from healthy volunteers. # (*P*<0.05) significant difference from patients without cytostatic treatment (unpaired *t* test).

Eryptosis is further induced by oxidative stress. Accordingly, reactive oxygen species (ROS) abundance was determined utilizing 2′,7′-dichlorodihydrofluorescein diacetate (DCFDA) fluorescence. As shown in Figure 7A and 7B, the DCFDA-fluorescence was significantly higher (by 20.9%) in erythrocytes freshly drawn from Group II, but not Group I, as compared to erythrocytes drawn from healthy volunteers. In order to test whether oxidative stress is induced by a blood-borne component, erythrocytes from healthy volunteers were incubated in plasma drawn from either, patients or healthy volunteers. As a result, the DCFDA fluorescence in erythrocytes exposed to plasma from Group II, but not Group I, was significantly higher (by 52.9 %) as compared to erythrocytes exposed to plasma from healthy volunteers (Figure 7C and 7D).

## DISCUSSION

The present study confirms that LC is paralleled by anemia. The anemia prevails despite enhanced reticulocyte numbers, indicating that the anemia is not primarily due to reduced formation of new erythrocytes. Instead, the anemia prevails obviously despite enhanced erythropoiesis. Presumably, the anemia leads to upregulation of erythropoietin, which in turn stimulates erythropoiesis [19]. Erythropoietin has previously been shown to inhibit eryptosis [20] but under the influence of high erythropoietin levels erythrocytes are apparently generated with relative high susceptibility to triggers of eryptosis [21]. In any case, the anemia thus appears to result from enhanced erythrocyte turnover. Anemia was previously shown to be an independent prognostic factor for the survival rate of cancer patients [22] and its presence is associated with decreased survival in almost all cancer types studied [23]. In severe cases, anemia may lead to hypoxia in specific organs, which might influence tumor behavior [24]. Tumor hypoxia is, in turn, associated with resistance to both chemotherapy and radiation therapy [25] and as well with increased angiogenesis, a marker of enhanced tumor aggressiveness [26].

The present observations point to a possible cause contributing to accelerated erythrocyte loss with development of anemia. The percentage of PS exposing erythrocytes was enhanced in blood from LC patients. PS at the erythrocyte surface fosters the phagocytosis of the affected erythrocytes, which are thus rapidly cleared from circulating blood [9]. Accordingly, the enhanced PS abundance at the erythrocyte surface could well contribute to the anemia of the LC patients with and without cytostatic treatment.

The enhanced PS exposure at the erythrocyte surface of LC patients under cytostatic treatment was paralleled by a slight, but significant increase of cell volume contrasting the typical decrease of cell volume during eryptosis. Cell shrinkage is usually triggered by increase of erythrocyte [Ca^2+^]_i_ resulting in activation of Ca^2+^ sensitive K^+^ channels, K^+^ exit, hyperpolarization of the cell membrane, Cl^−^ exit and thus cellular loss of KCl with osmotically obliged water [14]. [Ca^2+^]_i_ was not significantly different between erythrocytes isolated from LC patients and erythrocytes isolated from healthy volunteers. The lack of increased [Ca^2+^]_i_ provides an explanation for the absence of cell shrinkage.

The stimulation of cell membrane scrambling in LC patients may in part have been due to oxidative stress, a well known trigger of eryptosis [13]. An oxidant/antioxidant imbalance has been described in the blood of cancer patients [27]. Moreover, the increased cell membrane scrambling of erythrocytes from LC patients under cytostatic treatment is paralleled by increased abundance of ceramide at the erythrocyte surface. Ceramide is a well known stimulator of eryptosis [9]. It is noteworthy that enhanced ceramide formation predisposes to lung cancer [28]. The susceptibility to eryptosis is further enhanced by iron deficiency [9], which may play a key role in anemia of patients with malignancy [3]. In the present study, transferrin levels have not been measured and the contribution of iron deficiency to the observed stimulation of eryptosis thus remains uncertain.

Our data show that eryptosis in LC patients is further compounded by cytostatic treatment. Eryptosis could be triggered by a myriad of xenobiotics including several cytostatic drugs [29–43]. Interestingly, both topotecan and cisplatin used for cytostatic treatment of some patients included in this study, have been shown to trigger eryptosis *in vitro* [44, 45]. Notably, patients under platinum-based chemotherapy were shown to have higher risk for anemia as compared to patients with non-platinum based chemotherapy [46].

Besides inducing anemia, enhanced eryptosis could interfere with microcirculation, as PS-exposing erythrocytes bind to endothelial CXCL16/SR-PSO and thus adhere to the vascular wall [47]. Moreover, PS exposing erythrocytes trigger blood clotting and thrombosis [48]. As a matter of fact, cancer patients are at a higher risk for developing thrombosis [49], which, at least in theory, could be fostered by enhanced eryptosis associated with malignancy. Enhanced incidence of thrombosis has indeed been reported in LC patients [50].

In view of the present observations, inhibitors of eryptosis may ameliorate anemia in LC patients. The possibility must be kept in mind, however, that, at least in theory, inhibitors of suicidal erythrocyte death may similarly counteract apoptosis of tumor cells and thus interfere with cytostatic treatment. Correction of anemia would, on the other hand, be expected to lower release of erythropoietin, which has previously been shown to foster tumor cell proliferation, tumor angiogenesis and lymphangiogenesis [51].

In conclusion, lung cancer and its treatment using cytostatic drugs is paralleled by enhanced erythrocyte cell membrane scrambling with PS translocation to the erythrocyte surface, an effect presumably contributing to anemia in those patients.

## MATERIALS AND METHODS

### Patients, erythrocytes and treatments

Blood was drawn from patients suffering from LC (6 ♀, 11 ♂, age 52-85 years) and healthy volunteers (4 ♀, 11 ♂, age 41 - 70 years). The patients were recruited from the Department of Internal Medicine, University of Tübingen. The clinical characteristics of the patients are shown in Table 1. Six patients did not receive cytostatic therapy (Pat. Group I), whereas 11 patients received cytostatic treatment (Pat. Group II). Patients suffering from renal insufficiency or severe heart failure were excluded from the study. The study was approved by the ethics committee of the University of Tübingen (184/2003V) and has been performed in accordance with the Declaration of Helsinki. Both, patients and healthy volunteers provided written informed consent. In order to isolate the erythrocytes, whole blood was centrifuged at 120 x g for 20 min at 23°C and the platelets and leukocytes-containing supernatant was disposed. Measurements were made in freshly isolated erythrocytes or in erythrocytes (O^−^ blood group) from healthy young individuals incubated *in vitro* with 500 μl plasma from patients or healthy volunteers at a hematocrit of 0.4% for 24 hours. For all measurements 50,000 cells were counted.

### Annexin-V-binding and forward scatter

In order to determine annexin-V-binding, 2 μl of freshly drawn blood were mixed in 500 μl Ringer solution containing 5 mM CaCl_2_, subsequently stained with Annexin-V-FITC (1:200 dilution; ImmunoTools, Friesoythe, Germany) in Ringer solution containing 5 mM CaCl_2_ at 37°C for 15 min under protection from light. The annexin V abundance at the erythrocyte surface was subsequently determined on a FACS Calibur (BD, Heidelberg, Germany). Annexin-V-binding was measured with an excitation wavelength of 488 nm and an emission wavelength of 530 nm. A marker (M1) was placed to set an arbitrary threshold between annexin-V-binding cells and control cells. The same threshold was used for healthy erythrocytes and erythrocytes from lung cancer patients. A dot plot of forward scatter (FSC) vs. side scatter (SSC) was set to linear scale for both parameters.

### Reticulocyte count

For determination of the reticulocyte count, Lithium-Heparin-whole blood (2 μl) was added to 500 μl Retic-COUNT^®^ (thiazole orange) reagent from Becton Dickinson. Samples were stained for 30 min at room temperature in the dark and flow cytometry was performed according to the manufacturer's instructions. Forward scatter (FSC), side scatter (SSC) and thiazole orange-fluorescence intensity (in FL-1) of the blood cells were determined. The number of Retic-COUNT positive reticulocytes was expressed as the percentage of the total gated erythrocyte population. Gating of erythrocytes was achieved by analysis of FSC vs. SSC dot plots using CellQuest software.

### Confocal microscopy

For the visualization of eryptotic erythrocytes, 20 μl erythrocytes (1 × 10^6^ cells) were stained with Annexin V–FLUOS (1:100 dilution; Roche Diagnostics, Mannheim, Germany) in 200 μl Ringer solution containing 5 mM CaCl_2_. The erythrocytes were washed twice and finally resuspended in 200 μl Ringer solution containing 5 mM CaCl_2_. Forty μl were spread onto a glass slide and dried for 15 min on RT. The slides were covered with PROlong Gold antifade reagent (Invitrogen, Darmstadt Germany). Images were taken on a Zeiss LSM 5 EXCITER confocal laser-scanning microscope or with the phase light (Carl Zeiss MicroImaging, Germany) with a water immersion Plan-Neofluar 40/1.3 NA DIC. Scale bar 5 μm.

### Reactive oxygen species (ROS)

Oxidative stress was determined utilizing 2′,7′-dichlorodihydrofluorescein diacetate (DCFDA). 4 μl erythrocytes were mixed in 1 ml Ringer. From the resulting cell suspension, 150 μl were centrifuged (1600 rpm for 3 min at RT). Cells were stained with DCFDA (10 μM; Sigma, Schnelldorf, Germany) in Ringer solution at 37°C for 30 min and then washed three times in 150 μl Ringer solution. The DCFDA-loaded erythrocytes were resuspended in 200 μl Ringer solution and ROS-dependent fluorescence intensity was measured in FL-1 at an excitation wavelength of 488 nm and an emission wavelength of 530 nm on a FACS Calibur. Afterwards, the geomean of the ROS-dependent fluorescence was determined.

### Intracellular Ca^2+^

In order to quantify intracellular Ca^2+^, 2 μl of freshly drawn blood were mixed in 500 μl Ringer solution containing 5 mM CaCl_2_, stained with Fluo-3/AM (5 μM; Biotium, Hayward, USA) and incubated at 37°C for 30 min. Then Ca^2+^-dependent fluorescence intensity was measured in FL-1 with an excitation wavelength of 488 nm and an emission wavelength of 530 nm on a FACS Calibur. Subsequently, the geomean of the Ca^2+^ dependent fluorescence was determined.

### Ceramide formation

To determine ceramide abundance, a monoclonal antibody-based assay was used. 4 μl erythrocytes were mixed in 1 ml Ringer. From the resulting cell suspension, 100 μl were centrifuged (1600 rpm for 3 min at RT) and the erythrocytes were pelleted. Subsequently, cells were stained for 1 h at 37°C with 1 μg/ml anti-ceramide antibody (1:10 dilution; clone MID 15B4; Alexis, Grünberg, Germany) in phosphate-buffered saline (PBS) containing 0.1% bovine serum albumin (BSA). After two washing steps with 100 μl PBS-BSA, cells were stained for 30 min with polyclonal fluorescein-isothiocyanate (FITC)-conjugated goat anti-mouse IgG and IgM specific antibody (1:50 dilution; BD Pharmingen, Hamburg, Germany) in PBS-BSA. Unbound secondary antibody was removed by repeated washing with 50 μl PBS-BSA. The samples were resuspended in 200 μl PBS-BSA and then analyzed in FL-1 by flow cytometry at an excitation wavelength of 488 nm and an emission wavelength of 530 nm on a FACS Calibur. Finally, the geomean of the ceramide dependent fluorescence was determined.

### Statistics

Data are expressed as arithmetic means ± SEM. Mann-Whitney test or unpaired *t*-test was performed as appropriate to determine statistical significance between the two groups using GraphPad Prism version 6.00 for Windows, GraphPad Software, La Jolla California USA. For all correlations, Spearman nonparametric analysis was used. n denotes the number of individuals. *P*< 0.05 was considered significant.
